# Dietary patterns in Canadian men and women ages 25 and older: relationship to demographics, body mass index, and bone mineral density

**DOI:** 10.1186/1471-2474-11-20

**Published:** 2010-01-28

**Authors:** Lisa Langsetmo, Suzette Poliquin, David A Hanley, Jerilynn C Prior, Susan Barr, Tassos Anastassiades, Tanveer Towheed, David Goltzman, Nancy Kreiger, the CaMos Research Group

**Affiliations:** 1 CaMos National Coordinating Centre, McGill University, 687 Pine Ave W, Montreal, QC, H3A 1A1, Canada; 2 Department of Medicine, University of Calgary, 3330 Hospital Dr NW, Calgary, AB, T2N 4N1, Canada; 3 Department of Medicine, University of British Columbia, 2775 Laurel St, Vancouver, BC, V5Z 1M9, Canada; 4 Department of Human Nutrition, University of British Columbia, 2205 East Mall, Vancouver, BC, V5Z 1M9, Canada; 5 Department of Medicine, Queen's University, Etherington Hall, Kingston, ON, K7L 3N6, Canada; 6 Department of Medicine, McGill University, 687 Pine Ave W, Montreal, QC, Canada; 7 Dalla Lana School of Public Health, University of Toronto, 155 College St, Toronto, ON, Canada; 8 Cancer Care Ontario, 620 University Avenue, Toronto, ON M5G 2L7, Canada

## Abstract

**Background:**

Previous research has shown that underlying dietary patterns are related to the risk of many different adverse health outcomes, but the relationship of these underlying patterns to skeletal fragility is not well understood. The objective of the study was to determine whether dietary patterns in men (ages 25-49, 50+) and women (pre-menopause, post-menopause) are related to femoral neck bone mineral density (BMD) independently of other lifestyle variables, and whether this relationship is mediated by body mass index.

**Methods:**

We performed an analysis of 1928 men and 4611 women participants in the Canadian Multicentre Osteoporosis Study, a randomly selected population-based longitudinal cohort. We determined dietary patterns based on the self-administered food frequency questionnaires in year 2 of the study (1997-99). Our primary outcome was BMD as measured by dual x-ray absorptiometry in year 5 of the study (2000-02).

**Results:**

We identified two underlying dietary patterns using factor analysis and then derived factor scores. The first factor (nutrient dense) was most strongly associated with intake of fruits, vegetables, and whole grains. The second factor (energy dense) was most strongly associated with intake of soft drinks, potato chips and French fries, certain meats (hamburger, hot dog, lunch meat, bacon, and sausage), and certain desserts (doughnuts, chocolate, ice cream). The energy dense factor was associated with higher body mass index independent of other demographic and lifestyle factors, and body mass index was a strong independent predictor of BMD. Surprisingly, we did not find a similar positive association between diet and BMD. In fact, when adjusted for body mass index, each standard deviation increase in the energy dense score was associated with a BMD decrease of 0.009 (95% CI: 0.002, 0.016) g/cm^2 ^for men 50+ years old and 0.004 (95% CI: 0.000, 0.008) g/cm^2 ^for postmenopausal women. In contrast, for men 25-49 years old, each standard deviation increase in the nutrient dense score, adjusted for body mass index, was associated with a BMD increase of 0.012 (95% CI: 0.002, 0.022) g/cm^2^.

**Conclusions:**

In summary, we found no consistent relationship between diet and BMD despite finding a positive association between a diet high in energy dense foods and higher body mass index and a strong correlation between body mass index and BMD. Our data suggest that some factor related to the energy dense dietary pattern may partially offset the advantages of higher body mass index with regard to bone health.

## Background

The traditional approach to assessing the potential influence of diet is to determine the relationship of a particular nutrient to a given outcome after controlling for other nutrients. Another approach that gives complementary information is to identify underlying dietary patterns and determine the relationship of a particular pattern to a given outcome[1]. There is growing evidence that this second approach yields some strong and consistent predictors of multiple health outcomes [2-7]. Furthermore controlled trials have shown that it is possible to modify underlying dietary patterns[8,9].

Fractures related to osteoporosis contribute to increased mortality[10], lower quality of life [11], as well as substantial direct and indirect costs [12]. Secular changes in nutrition and lifestyle may contribute to an increased fracture burden if they are associated with bone fragility. Bone mineral density (BMD) is a strong predictor of fracture[13]. Certain nutrients, most notably calcium and vitamin D, are related to BMD[14]. The association between dietary patterns and BMD is less clear, although several studies have noted the potential benefit of fruits and vegetables [15-18]. A further complication is that while some dietary patterns might directly affect BMD, other effects may be dependent on intermediary changes in fat and/or lean mass.

The objective of the present analysis was to determine whether dietary patterns in men (ages 25-49, 50+) and women (pre-menopause, post-menopause) are related to BMD independently of other lifestyle variables and how the association is mediated by body mass index.

## Methods

### Subjects

We included participants in an on-going cohort study, the Canadian Multicentre Osteoporosis Study (CaMos) who completed the food frequency questionnaire with 10 or fewer missing responses in the food and drink section. A total of 9423 participants were enrolled. Among the 7315 participants who returned the food frequency questionnaire, 6539 had sufficiently complete data from the food frequency questionnaire to be included in this analysis.

The methodological details of the study have been described elsewhere [19]. Briefly, eligible participants were at least 25 years old at the beginning of the study, lived within a 50-kilometre radius of one of nine Canadian cities (St John's, Halifax, Quebec City, Toronto, Hamilton, Kingston, Saskatoon, Calgary, and Vancouver) and were able to converse in English, French, or Chinese (Toronto or Vancouver). Households were randomly selected from a list of residential phone numbers and participants were randomly selected from eligible household members using standard protocol. Of those selected, 42% agreed to participate in the study resulting in a baseline cohort of 9423 participants. Ethics approval was granted through McGill University and the appropriate research ethics boards for each participating centre. Signed informed consent was obtained from all study participants.

### Data collection

All participants were given a standardized interviewer-administered questionnaire (CaMos questionnaire^© ^1995) at baseline. The questionnaire covered demographics, health, nutrition, lifestyle, as well as a medical history that included both a detailed history of fracture and major risk factors for fracture. The questionnaire also included the Medical Outcomes Study 36-item Short Form (SF-36) with summary score for physical and mental components[20]. Medication and supplement use was assessed by a complete inventory of prescriptions and bottles brought to the interview. Baseline clinical assessment included height, weight, and BMD. Height was measured without shoes, using a height rod mounted on beam balance scale, a wall-mounted stadiometer, or ruler on the wall. Weight was measured in light clothing using a beam balance or electronic scale. A food frequency questionnaire was mailed to all participants in the second year of the study (1997-99). Follow-up visits were scheduled in the third year (1998-2000) for those between 40 and 60 years old and in the fifth year (2000-02) for all participants. These visits also included an interviewer-administered questionnaire, height and weight measurements, and BMD testing. Year 5 body mass index (kg/m^2^) and BMD were used as the main outcomes, as the Year 5 time point was the first clinical measurement after the food frequency questionnaire with no age exclusion criterion.

### Nutrition questionnaire

The self-administered nutrition questionnaire used in this study was based on the food frequency questionnaires developed and tested by Block (short form) and Willett [21,22], slightly modified for Canadian foods[23]. We note that energy intake estimates derived from the short Block questionnaire had a high correlation (r = 0.9) with energy intake estimates from the full Block questionnaire[21]. A total of 106 questions assessed use of nutritional supplements (n = 11), beverages (n = 18), foods (n = 51), condiments/fat (n = 7), summary items (n = 5), and change in diet over 20 years (n = 15) with food and beverage items selected from the Block FFQ. Each food and beverage item had a specified usual portion size as done in the Willett FFQ. Response options were one of nine mutually exclusive ordinal frequency categories ranging from never/less than once a month to 6 or more times per day. The FFQ has not been validated in an independent study.

### Bone mineral density

Bone mineral density was measured at the lumbar spine (L1-L4), femoral neck, trochanter, Ward's triangle, and total hip. Seven centres used Hologic densitometers and two centres used Lunar densitometers. All Lunar measurements were converted to equivalent Hologic values using standard reference formulas; this study required the formulas for femoral neck[24]. We did not use the lumber spine data in this analysis as degenerative changes and vertebral deformity both affect measurement at this site. Femoral neck was chosen as the reference hip site as it is used more frequently in the literature. All BMD values were adjusted to anthropomorphic phantoms that were scanned in each centre in the year of initial and all follow-up examinations.

### Statistical methods

The nutritional questionnaire was first screened for missing responses and those questionnaires with more than 10 missing responses in the food and beverage section were excluded. Missing responses for individual food items in the remaining questionnaires were replaced with the median response for the study sample [25]. Two variables were created to assess impact of imputing missing responses in further analyses: total number of missing responses and total difference between imputation using median intake and imputation using lowest intake. Total energy intake was based on frequency and specified portion size from the questionnaire together with caloric information from the Canadian Nutrient File[26].

After assessing the distributions and correlations between 69 food and beverage items, the responses were grouped into 34 categories (see Table 1 for list of summary categories). The total consumption for each category was determined by summing the monthly frequency of each item as measured by servings per month. The resulting variables were log transformed to adjust for skewness, and further rescaled to have mean of zero and standard deviation of 1. Factor analysis was performed using the 34 variables derived in the above fashion. Factor analysis is used to assess underlying patterns of variation and the derived factor score is a measure of common variation. Factor loading scores indicate the strength and direction of the association. Thus a high factor score indicates greater consumption of those foods with high factor loadings relative to with those with lower factor loadings.

**Table 1:** Food Categories and Factor Loadings Based on CaMos Year 2 Food Frequency Questionnaire

Category	Items	Factor Loadings	
		
		Nutrient dense	Energy dense
Coffee			

Tea			

Water	Bottled water, tap water	0.29	

Juice	Fresh fruit juice, frozen concentrated fruit juice	0.28	

Low fat milk	Skim and 1% milk	0.22	

High fat milk	2% and whole milk		0.21

Beer			0.26

Alcohol	Wine, liquor		

Soft drinks	Soft drinks, powdered drink mix		**0.42**

Fruit 1	Apples, oranges, bananas	**0.48**	

Fruit 2	Cantaloupe, other fruit	**0.54**	

Tomatoes	Tomatoes, tomato juice	**0.49**	

Green vegetables	Broccoli, spinach, other leafy greens	**0.61**	

Yellow vegetables	Cabbage, cauliflower, sweet potato, squash, brussel sprouts	**0.56**	

Other vegetables	Carrots, corn, peas, green beans, soup, other vegetables	**0.59**	

Whole grains	Dark whole grain bread, bran/granola, shredded wheat	**0.46**	

White bread	White bread/rolls	-0.24	0.37

Cereal	Cold cereal, cooked cereal	0.25	

Rice		0.26	

Pasta	Macaroni, spaghetti, noodles	0.22	0.26

Potatoes		0.25	0.24

Meat 1	Beef, pork, lamb, poultry (main dish or mixed)	0.28	0.28

Meat 2	Hamburger		**0.50**

Meat 3	Hotdog, lunch meat, smoked meat		**0.55**

Meat 4	Bacon, sausage		**0.56**

Fish	Fish (fresh, frozen, smoked, dried)	**0.40**	

Eggs			0.32

Cheese		0.25	0.30

Nuts	Nuts, peanut butter	0.21	0.20

Legumes	Beans, lentils, tofu, soybeans	0.37	

Sweets 1	Cake, pie, cookies		0.28

Sweets 2	Ice cream, chocolate, doughnuts		**0.47**

Added fats	Margarine, butter, mayonnaise		0.35

High fat potatoes	Fries, Potato chips		**0.58**

Percentage of overall variance explained by factor		9.3%	7.8%

Factor loadings with absolute value less than 0.2 indicated by blanks and those above 0.4 indicated in bold to visually emphasize strength of association. Sign of factor score indicates direction of association.

Models were run with different numbers of factors, and were assessed by an eigenvalue criterion where factors with eigenvalue less than 1 were dropped from consideration. Factors were rotated using varimax rotation to achieve uncorrelated factors with better interpretability.

A two-factor model resulted in factors that were both statistically important (eigenvalue > 1) and clinically relevant. Moreover, nearly identical factor loadings and factor scores were obtained when factor analysis with two factors was done separately for a randomly chosen sub-sample, for men, for women, and for those with/without imputed responses. The above stability of factor loadings across different subgroups was not present in models with 3 or more factors. The factor scores resulting from the analysis on the whole sample with two factors were used in all subsequent analyses. This choice enables between group comparisons.

It was hypothesized that behaviours would not be limited to a single domain and that other lifestyle variables would be associated with eating habits. Multiple linear regression was used to assess the relationship between factor scores and baseline variables, with factor scores being the dependent variable. The baseline variables considered were age, education (< 12 years schooling, high school diploma, post-secondary education), smoking (non-smoker, former smoker, current smoker), alcohol (non-drinker, moderate intake(< 1 drink/day in women, < 2 drink/day in men), high intake (1+ drink/day in women, 2+ drink/day in men))), activity (kcalorie per week spent on moderate activity, vigorous work, or strenuous sports calculated from weekly inventory of activities in these three categories), sedentary time (time per day spent sitting or sleeping), daily milk consumption, daily use of supplements (vitamin D, calcium).

It was hypothesized that there would be a relationship between diet and BMD and that the relationship of diet to BMD would be partially mediated by body mass index. We assessed this hypothesis using a series of regression models testing both direct and indirect associations. The first set of models used body mass index as the dependent variable; the second set of models used femoral neck BMD as the dependent variable, adjusting for height but not for body mass index; and the final set of models used femoral neck BMD as the dependent variable, adjusting for both height and body mass index. We also looked at the direct association between body mass index and BMD using univariate regression. All regression analyses were *a priori *stratified by sex, and by age category in men and menopausal status in women based on known differences in both diet and bone mineral metabolism. All models included *a priori *specified potential confounders including all baseline variables listed above, together with study centre, medication (antiresorptives, corticosteroids), oophorectomy, and recent menopause (last 5 years); as relevant. No adjustments were made for multiple comparisons. In addition to regression diagnostics, we used robust regression to determine whether regression was sensitive to extreme values and found only minor differences not impacting overall interpretation.

## Results

The baseline characteristics of the 1928 men and 4611 women in the study sample are shown in Table 2. Our analyses excluded 956 men and 1928 women because the Year 2 food frequency questionnaire was missing or incomplete. Men who were excluded were on average 3.1 (95% CI: 1.9, 4.3) years older, had lower SF-36 physical health and mental health scores (mean difference 3.1 (95% CI: 2.3, 3.9) and 1.2 (95% CI: 0.6, 1.9) respectively), but had similar body mass index and total hip BMD compared with those in the study. Women who were excluded were on average 6.2 (95% CI: 5.6, 6.9) years older, had lower SF-36 physical health and mental health scores (mean difference 3.9 (95% CI: 3.4, 3.6) and 0.9 (95% CI: 0.4, 1.4) respectively), had 0.032 (95% CI: 0.024, 0.039) g/cm^2 ^lower femoral neck BMD, but had similar body mass index compared with those in the study.

**Table 2:** Baseline Characteristics of the Study Sample

	Men (N = 1928)		Women (N = 4611)	
	**Mean**	**SD**	**Mean**	**SD**

Age (years)	58.8	13.5	61.2	12.2

Height (cm)	174.1	7.1	160.1	6.4

Weight (kg)	82.2	13.7	69.1	13.8

Body mass index (kg/m^2^)	27.1	4.0	26.9	5.1

Femoral neck BMD (g/cm^2^)	0.81	0.13	0.72	0.12

SF-36 physical^a^	49.9	8.6	47.6	10.0

SF-36 mental^a^	54.5	7.7	53.3	8.8

Sedentary time^b ^(hours/day)	14.6	3.1	13.9	3.0

Activity^c^(1000 kcal/week)	5.8	5.1	4.4	3.2

	N	%	N	%

Current smoker	300	15.6	630	13.7

Current alcohol use	1479	76.7	2754	59.7

Antiresorptives	5	0.3	1317	28.6

Corticosteroids	178	9.2	623	13.5

Vitamin D from supplements	338	17.5	1551	33.6

Calcium from supplements	659	34.2	2574	55.8

Caucasian (white)	1822	94.5	4438	96.2

Chinese	54	2.8	80	1.7

North American Indian	24	1.2	56	1.2

South Asian	15	0.8	18	0.4

^a ^Scores range from 0 to 100 with 0 indicating worst possible health and 100 indicating best possible health

^b ^Total time spent sitting or sleeping

^c ^Moderate, strenuous or vigorous activity

The two factors and the corresponding factor loadings are shown in Table 1. The first factor (nutrient dense) was most strongly associated with intake of fruits, vegetables, and whole grains. The second factor (energy dense) was most strongly associated with intake of soft drinks, potato chips and French fries, certain meats (hamburger, hot dog, lunch meat, smoked meat, bacon, and sausage), and certain desserts (doughnuts, chocolate, ice cream). The items listed above all had positive loading with the dietary patterns, indicating that above average intake contributed to a positive score on the associated factor and below average intake contributed to a negative score. The distributions of both factor scores were approximately normal with mean = 0 and standard deviation = 0.9.

Both factor scores were related to sex, but in opposite directions. The mean score for the nutrient dense factor was 0.43 (95% CI: 0.37-0.48) standard deviations higher in women than men. The mean score for the energy dense factor was 0.52 (95% CI: 0.46-0.57) standard deviations lower in women than men. The results of multivariate models predicting nutrient dense and energy dense factor scores in men and women by demographic and lifestyle variables are shown in Table 3. Age was a strong linear predictor, with younger participants having on average lower nutrient dense scores and higher energy dense scores than older participants among both men and women.

**Table 3:** Regression Coefficients for Baseline Variables as Predictors of Dietary Factor Scores in Men and Women from Multivariate Model

Independent variables (Baseline)		Outcome variables (Year 2)			
		
		Nutrient dense **score **^a^		Energy dense **score**^a^	
		
		Men	Women	Men	Women
Age (10 years)		**0.20** **0.16, 0.23**	**0.15** **0.13, 0.17**	**-0.15** **-0.19, -0.12**	**-0.13** **-0.15, -0.11**

Education ^b^	< 12 years	**-0.24** **-0.36, -0.13**	**-0.20** **-0.27, -0.14**	-0.01 -0.13, 0.12	0.05 -0.01, 0.11
	
	Post-secondary	**0.30** **0.19, 0.40**	**0.26** **0.19, 0.33**	**-0.13** **-0.24, -0.02**	**-0.09** **-0.15, -0.02**

Smoking ^c^	Current	**-0.27** **-0.40, -0.13**	**-0.36** **-0.44, -0.28**	**0.39** **0.24, 0.53**	**0.25** **0.17, 0.33**
	
	Former	-0.02 -0.12, 0.08	0.02 -0.04, 0.08	**0.13** **0.02, 0.23**	0.00 -0.06, 0.06

Alcohol ^d^	Moderate	**0.26** **0.15, 0.36**	**0.21** **0.15, 0.26**	**0.33** **0.21, 0.44**	**0.12** **0.06, 0.18**
	
	High	0.14 -0.05, 0.32	**0.14** **0.02, 0.26**	**0.50** **0.30, 0.69**	**0.27** **0.15, 0.39**

Activity ^e^ (2500 kcal/week)		**0.05** **0.03, 0.07**	**0.04** **0.02, 0.06**	**0.03** **0.01, 0.05**	**0.04** **0.02, 0.06**

Sedentary time ^f^ (hours/day)		0.00 -0.01, 0.02	-0.01 -0.02, 0.00	**0.02** **0.00, 0.03**	**0.02** **0.01, 0.03**

Milk consumption (250 mL/day)		**0.03** **0.00, 0.06**	**0.07** **0.05, 0.09**	0.03 0.00, 0.06	0.00 -0.02, 0.03

Calcium from supplements (500 mg/day)		0.06 -0.02, 0.14	**0.03** **0.00, 0.07**	0.02 -0.06, 0.11	-0.03 -0.06, 0.00

Vitamin D from supplements (200 IU/day)		**0.08** **0.03, 0.13**	0.03 0.00, 0.05	-0.04 -0.10, 0.01	**-0.04** **-0.06, -0.01**

^a ^Outcome variables are unitless but standardized to have mean = 0 and SD = 1. Each individual's diet was characterized by both a nutrient dense score and an energy dense score. A positive regression coefficient for the nutrient dense score indicates category or increase in the independent variable is associated with a greater consumption of fruits, vegetables and whole grains relative to other foods. A positive regression coefficient for the energy dense scores indicates category or increase in the independent variable is associated with a greater consumption of chips/fries, processed meat, soft drinks, and certain desserts relative to other foods.

Reference or comparison category:

^b ^Reference category = high school diploma.

^c ^Reference category = never-smoker

^d ^Reference category = non-drinker,

^e ^Moderate, strenuous or vigorous activity

^f ^Total time spent sitting or sleeping

Confidence intervals not crossing 0 prior to rounding are indicated in bold type (equivalent to *p *< 0.05)

Higher educational attainment, vitamin D supplement use, and non-smoking were independently associated with higher nutrient dense scores and lower energy dense scores. Higher alcohol intake and greater physical activity were both independently associated with higher nutrient dense scores and higher energy dense scores. Finally, higher milk consumption and greater use of calcium supplements was associated with a higher nutrient dense score and longer sedentary time was associated with a higher energy dense score.

We assessed the relationship of total energy intake to the factor scores. Both factor scores were positively correlated with energy intake (data not shown). Since the factor scores were derived using log-transformed variables these relationships were not linear. The Pearson correlation of the log-transformed energy intake and the factor scores was *r *= 0.54 for the nutrient dense factor score and *r *= 0.49 for the energy dense factor score. Furthermore, among those with a given energy intake there was an inverse relationship between the nutrient dense factor score and the energy dense factor score. In view of these strong correlations, we performed a secondary analysis including the difference between the two factor scores and the log transformed energy intake as adjustment factor. The difference was calculated as the energy dense factor score minus the nutrient dense factor score and can be interpreted as the direct comparison between a diet high in energy dense foods versus a diet high in nutrient dense foods.

**Figure 1:**
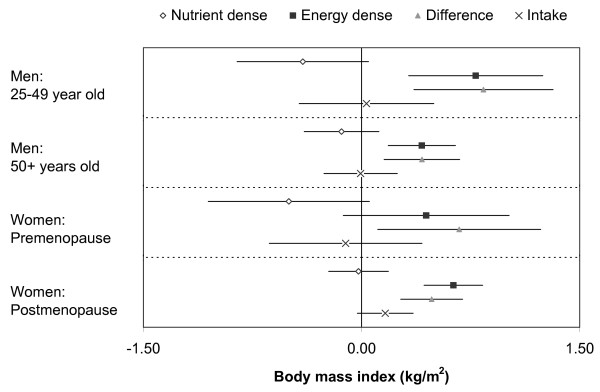
**Regression coefficients for dietary patterns and energy intake as predictors of body mass index**. The parameter estimates are for each 1 SD increase of the nutrient dense factor score, the energy dense factor score, the difference between energy dense and nutrient dense factor score, and the log-tranformed energy intake (1 SD is roughly 36% change in energy intake). P-values for null hypothesis (from top to bottom) Younger Men: 0.080, 0.001, 0.001, 0.884; Older Men: 0.294, < 0.001, 0.002, 0.961; Premenopausal Women: 0.077, 0.126, 0.019, 0.683; Postmenopausal Women: 0.842, < 0.001, < 0.001, 0.096. Analyses were run for the two factor scores and for the difference between factor scores and energy intake separately due to multicollinearity between intake and factor scores. All models are adjusted for age, height, center, education, smoking, alcohol consumption, activity, sedentary time, milk consumption, supplements (vitamin D, calcium); and antiresorptives, corticosteroids, recent (< 5 years) menopause, oophorectomy, as relevant. A high nutrient dense score indicates a greater consumption of fruits, vegetables and whole grains relative to other foods, a high energy dense scores indicates a greater consumption of chips/fries, processed meat, soft drinks, and certain desserts relative to other foods. A high difference indicates more energy dense food relative to nutrient dense foods.

The estimated parameters for factor scores, difference between scores, and energy intake as predictors of body mass index in each of the four groups (men 25-49 years, men 50+ years, women pre-menopause, women post-menopause) are shown in Figure 1. Higher nutrient dense factor scores were associated with a similar or lower body mass index. Higher energy dense factor scores were associated with a higher body mass index. The difference between scores (energy dense score-nutrient dense score) was positively associated with body mass index, but energy intake was not associated with body mass index. There was no evidence of between group heterogeneity of the regression coefficients as assessed by analysis of variance (p-values between 0.24 to 0.75). The above associations were adjusted for age, height, study centre, education, smoking, alcohol, activity, sedentary time, milk consumption, supplements (vitamin D and calcium), antiresorptive therapy, corticosteroids, oophorectomy, and recent menopause (final menstrual flow within the last 5 years).

**Figure 2:**
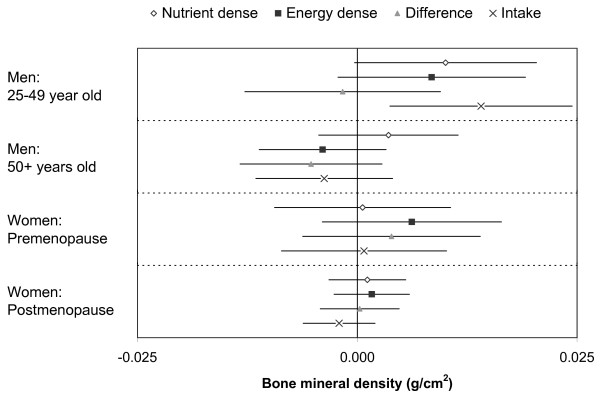
**Regression coefficients for dietary patterns and energy intake as predictors of femoral neck BMD without adjustment for body mass index**. The parameter estimates are for each 1 SD increase of the nutrient dense factor score, the energy dense factor score, the difference between energy dense and nutrient dense factor score, and the log-tranformed energy intake (1 SD is roughly 36% change in energy intake). P-values for null hypothesis (from top to bottom) Younger Men: 0.057, 0.120, 0.770, 0.008; Older Men: 0.381, 0.284. 0.202, 0.357; Premenopausal Women: 0.907, 0.232, 0.449, 0.874; Postmenopausal Women: 0.607. 0.451, 0.905, 0.324.

**Figure 3:**
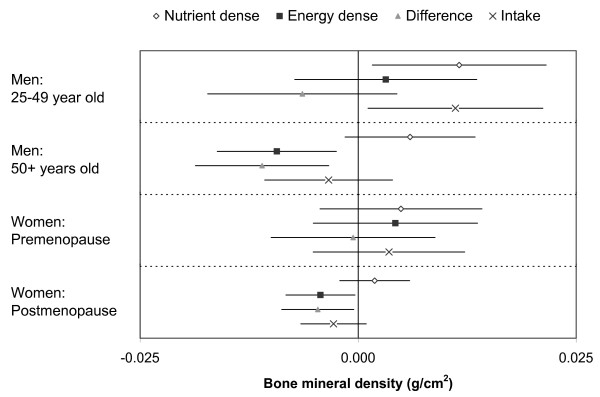
**Regression coefficients for dietary patterns and energy intake as predictors of femoral neck BMD with adjustment for body mass index**. The parameter estimates are for each 1 SD increase of the nutrient dense factor score, the energy dense factor score, the difference between energy dense and nutrient dense factor score, and the log-tranformed energy intake (1 SD is roughly 36% change in energy intake). P-values for null hypothesis (from top to bottom) Younger Men: 0.028, 0.552, 0.258, 0.030; Older Men: 0.118, 0.007, 0.005, 0.399; Premenopausal Women: 0.300, 0.374, 0.904, 0.425; Postmenopausal Women: 0.353, 0.032, 0.028, 0.110.

The estimated parameters for factor scores, difference between scores, and energy intake as predictors of BMD adjusted for the same confounders noted above are shown in Figure 2 (no adjustment for BMI) and Figure 3 (with adjustment for BMI). The associations between the factor scores and BMD without adjusting for body mass index were not consistent across subgroups and did not follow the previously noted relationships between dietary factors and body mass index. There was some unexplained between group heterogeneity of the regression coefficients (p-values between 0.03 and 0.46). There was a positive association between energy intake and femoral neck BMD among young men, but very weak and not statistically significant associations in all other subgroups.

There was a strong positive correlation between body mass index and femoral neck BMD in all subgroups, both in univariate and multivariate analyses. As a result of the serial correlations, the association between the nutrient dense factor score and BMD was more positive and the association between the energy dense factor score and BMD was more negative in each subgroup in the analysis adjusted for body mass index compared with the unadjusted analysis. The between group heterogeneity in the body mass index adjusted analysis was slightly less than that of the unadjusted analysis (p-values between 0.06 and 0.31).

## Discussion

We identified two dietary patterns (nutrient dense and energy dense) in Canadian men and women analogous to dietary patterns ("Prudent" and "Western") noted in other studies [2-5]. Surprisingly, we did not find any consistent relationship between diet and BMD without adjustment for body mass index, and among postmenopausal women, this association if present was very small. Genetics and early environment play a strong role in the development of peak bone mass and genetics may also impinge on the rate of bone loss[27]. Later environmental determinants may have an effect on the rate of bone loss, but these effects may be small in comparison relative to other determinants of BMD. The early determination of overall bone mass may explain the overall very weak associations between diet and BMD despite the noted relationship between diet and body mass index.

We posit there may be an association between the increased consumption of nutrient dense foods relative to energy dense foods and BMD after further adjustment for body mass index. After adjustment for body mass index, higher intake of nutrient dense food was associated with a higher BMD among men ages 25-49. Weaker but still positive relationships were found among older men and women, but none of these results were statistically significant. A higher energy dense factor score, adjusted for body mass index, was associated with lower BMD among men ages 50 and over, and post-menopausal women. Albeit non-significant, the reverse association was found among younger men and women in our study, but it is not clear whether this was a reflection of overall uncertainty or true heterogeneity. Viewing these results together suggests a comparative advantage of nutrient dense foods over energy dense foods, except among premenopausal women. In contrast to the null results for pre-menopausal women, Okubo et al., using a factor analysis approach similar to the analysis we have used in this paper, found that a dietary pattern including fish, fruits, and vegetables and low in meat and processed meat was associated with higher BMD in pre-menopausal Japanese farm women [28]. Components of the nutrient dense dietary pattern, notably fruits and/or vegetables, have also been shown to be associated with BMD [15-18].

The consistency of dietary patterns between our study and other studies enable better between-study comparisons, and suggests that our results may be applicable to different populations. We found that the factor scores were related to several demographic factors, including sex, age, and education. The lower nutrient dense scores and higher energy dense scores among younger age groups is alarming from the view of population health, given the relationships found between "Prudent" and "Western" dietary patterns and adverse health outcomes including coronary heart disease and stroke [2-5].

Comparison of our results with studies based on assessment of nutrients is more difficult. The energy dense dietary pattern included items high in carbohydrates, high in fat, and high in both. It has been previously noted that dietary patterns high in sweets are associated with lower BMD[18], however the impact of dietary fat is unclear. Thus, one study demonstrated a negative association between intake of unsaturated fat and BMD, and a positive association between intake of saturated fat and BMD[29]. In contrast, another study found a negative association between intake of saturated fat and BMD[30]. The observed associations may depend on complex interactions between fat and other nutrients and an analysis of these interactions would depend both on identification of the nutrients involved and proper model specification.

We confirmed the hypothesis that diet was a predictor of body mass index. Notably, we found that a higher intake of energy dense foods relative to nutrient dense foods, as seen directly by use of the difference score, was associated with increased body mass index across all subgroups. In contrast, there was no association between overall energy intake and body mass index. Energy intake is related to energy density of foods consumed, and increasing the energy density of a diet has been shown to increase total energy intake [31-33]. Some of the foods associated with the nutrient dense factor have low energy density, notably fruits and vegetables[6,8]. Therefore, the observed association between dietary patterns and body mass index may be causally linked by increased energy intake without a concomitant increase in energy expenditure. Assessment of this causal path is problematic in observational studies since it is difficult to measure energy balance with sufficient accuracy[34]. Metabolic efficiency is usually unknown and typical measures of intake and activity are susceptible to both bias and error. The most probable source of bias in this case was the use of a standard portion size for all food items. Underestimation of portion size would lead to greater underestimation in the calculation of total energy intake for those who consumed more energy dense foods. This mechanism could introduce sufficient bias to mask any association between energy intake and body mass index.

We also confirmed the hypothesis that body mass index was strongly associated with BMD. This is partially attributable to the fact that those with larger bone size have both greater body mass index and higher BMD. Body mass index is also associated with both lean and fat body mass, which are also predictors of bone mineral content[35].

The strengths of our study include the fact that we were able to assess lifestyle and demographics, including dietary patterns, together with measured assessment of body mass index and BMD values in a large randomly selected population, including both men and women over a wide age range. This allowed us to study the relationship between diet and BMD after adjusting for many of the variables that are related to dietary patterns. Our use of dietary patterns takes into account interactions between nutrient and foods not possible using the single nutrient approach.

This study has limitations. The factor analysis used is exploratory in nature and involves decisions that are subjective. Not all members of the cohort completed the food frequency questionnaire nor had BMD assessed at year 5. Those with a poor diet might have had a missing FFQ or might have died before second BMD measurement, with bias most likely toward the null. The limited scope and specified portion size of the FFQ may yield biased estimates of absolute energy intake. Some ethnic groups included in the study (e.g. Chinese) may have had dietary habits not adequately captured by the food frequency questionnaire. Furthermore, under-representation of ethnic minorities in the study may limit generalizability. Finally, we cannot rule out the possibility of residual confounding since dietary patterns may be related to other unmeasured health behaviours.

## Conclusions

In summary, we found no consistent relationship between diet and BMD despite finding a positive association between a diet high in energy dense foods and low in nutrient dense foods and higher body mass index. Because body mass index is strongly associated with BMD it was expected that similar associations would be true for diet and BMD. There may be associations between dietary patterns and BMD after adjusting for body mass index, which partially offset the expected positive effect of body mass index on BMD.

## Competing interests

David Hanley, MD; advisory board, honoraria, grants: Abbott Laboratories, Amgen, Eli Lilly, Merck, Novartis, Proctor & Gamble, sanofi-aventis, Servier, Wyeth-Ayerst, Nycomed, Paladin. Susan Barr, PhD; consulting: International Dairy Foods Association Tassos Anastassiades, MD; honoraria: Merck, Proctor & Gamble, Schering Plough, Servier Tanveer Towheed, MD; honoraria, grants: Abbott Laboratories, Bristol-Myers Squib, Novartis, sanofi-aventis David Goltzman, MD; consulting: Eli Lilly, Novartis, Merck-Frosst, Proctor & Gamble, sanofi-aventis, Servier Lisa Langsetmo, Suzette Poliquin, Jerilynn Prior and Nancy Kreiger have no competing interests.

## Authors' contributions

LL, SP, DAH, JCP, TA, TT, and NK worked on the study design. LL, SP, NK performed the data analysis. LL, SP, JCP, SB, DG, NK worked on the interpretation of results. All authors were involved in drafting and revising the manuscript and have read and approved the final manuscript. Other members of the CaMos Research Group were involved in the initial study design, recruitment of participants, data collection, quality control, review of projects, retention of the cohort, and other projects.

## Pre-publication history

The pre-publication history for this paper can be accessed here:

http://www.biomedcentral.com/1471-2474/11/20/prepub
